# Supplementary material for: Modelling Skylarks (Alauda arvensis) to Predict Impacts of Changes in Land Management and Policy: Development and Testing of an Agent-Based Model
Source: PLoS One. 2013 Jun 6;8(6):e65803. doi: 10.1371/journal.pone.0065803 (PMC3675089; doi:10.1371/journal.pone.0065803)
Supplement: Supporting Information S4 — The skylark ODdox as a zipped archive. (ZIP) [file pone.0065803.s004.zip › Skylark_ODdox/pesticide_8cpp.html]

ALMaSS Skylark ODdox: pesticide.cpp File Reference


ALMaSS Skylark ODdox
 2.0


- Main Page
- Related Pages
- Classes
- Files

- File List
- File Members

Macros |
Variables

pesticide.cpp File Reference

`#include <vector>`  
`#include <cstdio>`  
`#include <math.h>`  
`#include "ls.h"`

|  |  |
| --- | --- |
| Macros | |
| #define | \_CRT\_SECURE\_NO\_DEPRECATE |
| #define | CFG\_CHANNEL\_BITS   8 |
| #define | CFG\_CHANNEL\_MAXVAL   (2^CFG\_CHANNEL\_BITS-1) |
| #define | SV\_INT32   int |
| #define | SV\_INT8   char |
| #define | SV\_UINT32   unsigned int |
| #define | SV\_UINT8   unsigned char |

|  |  |
| --- | --- |
| Variables | |
| CfgInt | cfg\_pest\_productapplic\_enddate ("PEST\_PRODUCTAPPLIC\_ENDDATE", CFG\_CUSTOM, 1) |
| CfgInt | cfg\_pest\_productapplic\_period ("PEST\_PRODUCTAPPLIC\_PERIOD", CFG\_CUSTOM, 1) |
| CfgInt | cfg\_pest\_productapplic\_startdate ("PEST\_PRODUCTAPPLIC\_STARTDATE", CFG\_CUSTOM,-1) |
| CfgInt | cfg\_pest\_productapplic\_startdate2 ("PEST\_PRODUCTAPPLIC\_STARTDATE\_TWO", CFG\_CUSTOM,-1) |
| CfgBool | cfg\_pest\_residue\_or\_rate ("PEST\_RESIDUE\_OR\_RATE", CFG\_CUSTOM, true) |
| class Pesticide \* | g\_pest |
| static CfgFloat | l\_pest\_ai\_half\_life ("PEST\_AI\_HALF\_LIFE", CFG\_CUSTOM, 10.0) |
| CfgFloat | l\_pest\_daily\_mort ("PEST\_DAILY\_MORTALITY", CFG\_CUSTOM, 0.25) |
| static CfgInt | l\_pest\_diffusion\_grid\_count ("PEST\_DIFFUSION\_GRID\_COUNT", CFG\_CUSTOM, 1) |
| static CfgFloat | l\_pest\_diffusion\_slope ("PEST\_DRIFT\_SLOPE", CFG\_CUSTOM,-0.6122) |
| CfgBool | l\_pest\_enable\_pesticide\_engine ("PEST\_ENABLE\_PESTICIDE\_ENGINE", CFG\_CUSTOM, false) |
| CfgFloat | l\_pest\_insecticide\_amount ("PEST\_INSECTICIDE\_AMOUNT", CFG\_CUSTOM, 0.00) |
| CfgFloat | l\_pest\_product\_0\_amount ("PEST\_PRODUCT\_ZERO\_AMOUNT", CFG\_CUSTOM, 0.004145) |
| CfgFloat | l\_pest\_product\_1\_amount ("PEST\_PRODUCT\_ONE\_AMOUNT", CFG\_CUSTOM, 0.004145) |
| CfgBool | l\_pest\_use\_application\_rate ("PEST\_USE\_APPLICATIONRATE", CFG\_CUSTOM, false) |
| static CfgFloat | l\_pest\_zero\_threshold ("PEST\_ZERO\_THRESHOLD", CFG\_CUSTOM, 0.00001) |

---

## Macro Definition Documentation

#define \_CRT\_SECURE\_NO\_DEPRECATE

#define CFG\_CHANNEL\_BITS   8

#define CFG\_CHANNEL\_MAXVAL   (2^CFG\_CHANNEL\_BITS-1)

#define SV\_INT32   int

#define SV\_INT8   char

#define SV\_UINT32   unsigned int

Referenced by Pesticide::SavePPM().

#define SV\_UINT8   unsigned char

Referenced by Pesticide::SavePPM().

---

## Variable Documentation

CfgInt cfg\_pest\_productapplic\_enddate("PEST\_PRODUCTAPPLIC\_ENDDATE", CFG\_CUSTOM, 1)

CfgInt cfg\_pest\_productapplic\_period("PEST\_PRODUCTAPPLIC\_PERIOD", CFG\_CUSTOM, 1)

CfgInt cfg\_pest\_productapplic\_startdate("PEST\_PRODUCTAPPLIC\_STARTDATE", CFG\_CUSTOM,-1)

CfgInt cfg\_pest\_productapplic\_startdate2("PEST\_PRODUCTAPPLIC\_STARTDATE\_TWO", CFG\_CUSTOM,-1)

Referenced by Orchard::DoDevelopment(), and OrchardBand::DoDevelopment().

CfgBool cfg\_pest\_residue\_or\_rate("PEST\_RESIDUE\_OR\_RATE", CFG\_CUSTOM, true)

class Pesticide\* g\_pest

Referenced by Orchard::DoDevelopment(), OrchardBand::DoDevelopment(), Landscape::Landscape(), Farm::ProductApplication0(), Farm::ProductApplication1(), Landscape::SupplyPesticide(), Landscape::Tick(), and Landscape::~Landscape().

CfgFloat l\_pest\_ai\_half\_life("PEST\_AI\_HALF\_LIFE", CFG\_CUSTOM, 10.0)

static

Referenced by Pesticide::Pesticide().

CfgFloat l\_pest\_daily\_mort("PEST\_DAILY\_MORTALITY", CFG\_CUSTOM, 0.25)

CfgInt l\_pest\_diffusion\_grid\_count("PEST\_DIFFUSION\_GRID\_COUNT", CFG\_CUSTOM, 1)

static

Referenced by Pesticide::DiffusionMaskInit(), and Pesticide::DiffusionMaskInitTest().

CfgFloat l\_pest\_diffusion\_slope("PEST\_DRIFT\_SLOPE", CFG\_CUSTOM,-0.6122)

static

Referenced by Pesticide::DiffusionFunction().

CfgBool l\_pest\_enable\_pesticide\_engine("PEST\_ENABLE\_PESTICIDE\_ENGINE", CFG\_CUSTOM, false)

Referenced by Pesticide::Pesticide(), Pesticide::Tick(), and Pesticide::~Pesticide().

CfgFloat l\_pest\_insecticide\_amount("PEST\_INSECTICIDE\_AMOUNT", CFG\_CUSTOM, 0.00)

CfgFloat l\_pest\_product\_0\_amount("PEST\_PRODUCT\_ZERO\_AMOUNT", CFG\_CUSTOM, 0.004145)

Referenced by Farm::ProductApplication0().

CfgFloat l\_pest\_product\_1\_amount("PEST\_PRODUCT\_ONE\_AMOUNT", CFG\_CUSTOM, 0.004145)

Referenced by Farm::ProductApplication1().

CfgBool l\_pest\_use\_application\_rate("PEST\_USE\_APPLICATIONRATE", CFG\_CUSTOM, false)

Referenced by Pesticide::TwinMapSpray().

CfgFloat l\_pest\_zero\_threshold("PEST\_ZERO\_THRESHOLD", CFG\_CUSTOM, 0.00001)

static

Referenced by Pesticide::DiffusionFunction(), and Pesticide::MainMapDecay().


- CJT
- MSVC
- ALMaSS Working Source
- Landscape
- pesticide.cpp
- Generated on Thu Jan 10 2013 13:15:35 for ALMaSS Skylark ODdox by
   1.8.1.1
